# Endostrat: a multicenter study of AI-assisted two-level risk stratification for gastric precancerous and early neoplastic lesion screening

**DOI:** 10.3389/fmed.2026.1918150

**Published:** 2026-08-28

**Authors:** Zian Chen, Huihui Ma, Chenxi Yang, Hongwei Hao, Qian Gu, Luwei Jia, Zhixu Lu, Ruoying Ding, Renzhong Li, Linghui Song, Zhijie Feng

**Affiliations:** 1 Department of Gastroenterology, The Second Hospital of Hebei Medical University, Hebei Key Laboratory of Gastroenterology, Hebei Institute of Gastroenterology, Hebei Clinical Research Center for Digestive Diseases, Shijiazhuang, Hebei, China; 2 Cangzhou Central Hospital, Cangzhou, Hebei, China; 3 Neiqiu County People’s Hospital, Xingtai, Hebei, China; 4 Hebei Medical University, Shijiazhuang, Hebei, China

**Keywords:** artificial intelligence, deep learning, endoscopy, gastric cancer screening, multiple instance learning, risk stratification

## Abstract

**Background:**

In large-scale endoscopic screening, neoplastic lesions constitute only a small fraction of the cases examined, making detection susceptible to reader fatigue. Most existing AI systems rely on image-level annotations and single-stage classification, limiting patient-level assessment and safety-aware review prioritization. We developed and validated EndoStrat, a two-stage AI-assisted risk stratification system that triages low-risk patients and prioritizes high-risk patients for focused endoscopic review.

**Methods:**

This retrospective, multicenter study included 8,829 patients (approximately 220,000 white-light endoscopic images) from three centers with distinct pathological compositions. Patients were classified into four categories: normal/superficial gastritis, atrophic gastritis/intestinal metaplasia (Atrophy/IM), low-grade intraepithelial neoplasia (LGIN), and high-grade intraepithelial neoplasia/early gastric cancer (HGIN/EGC). Center 1 was split into a training cohort (*n* = 3,555) and an internal validation cohort (*n* = 890); Center 2 (*n* = 2,896) and Center 3 (*n* = 1,488) served as independent external validation cohorts. EndoStrat employs a Level-1 screening module that triages patients into low-risk and review-required groups using patient-level weak supervision and a Level-2 stratification module that outputs a continuous risk score integrating image features with age and sex for priority-based review. Two comparison methods—an image-level classifier and a four-class multiple instance learning classifier—were evaluated under identical conditions.

**Results:**

Across the three validation cohorts, Level 1 achieved AUCs of 0.770, 0.720, and 0.674, which were numerically higher than those of both comparators. At the exact fixed operating threshold of 0.22, the HGIN/EGC flag rate was 93.0% or higher in all cohorts, and the neoplasia miss rate ranged from 5.8% to 8.1%. Among patients with normal/superficial pathology, 21.1%–26.1% were classified as low risk; across all patients, the corresponding overall low-risk assignment rates ranged from 11.2% to 19.6%. In a module-level analysis including all patients with pathology other than Normal/Superficial irrespective of Level-1 classification, the Level-2 continuous risk score showed a clear monotonic gradient across Atrophy/IM, LGIN, and HGIN/EGC (all *p* < 0.001), with concordance indices of 0.907–0.967 after clinical features were incorporated.

**Conclusions:**

EndoStrat showed consistent risk-stratification performance across three validation cohorts with different pathological compositions. These retrospective findings suggest that EndoStrat may have potential to support risk-based review prioritization while retaining a high proportion of neoplastic cases in the review-required group. Prospective multicenter studies are needed to determine its effects on clinical workflow, diagnostic performance, and patient outcomes.

## Introduction

Gastric cancer is among the most common malignancies worldwide, with approximately 970,000 new cases annually, and it is the fifth leading cause of cancer-related death; its incidence is particularly high in East Asia (1, 2). Early detection is critical for improving prognosis: the 5-year survival rate for early gastric cancer (EGC) can exceed 90%, whereas that for advanced gastric cancer is approximately 10%–30% (2). However, in large-scale screening, individuals with neoplastic lesions constitute only a small fraction of those examined. Endoscopists must identify scattered abnormal cases among a vast number of normal images—an inherently low-prevalence detection task that readily leads to reader fatigue and delayed identification of clinically important cases (3, 4). Accordingly, optimizing review prioritization while maintaining diagnostic safety is a central challenge for improving screening efficiency (4, 5).

In recent years, artificial intelligence (AI)-assisted endoscopic diagnosis has advanced substantially (5, 6). Multiple studies have shown that deep learning-based systems achieve high accuracy in gastric mucosal lesion detection and histopathological classification, and several systems (e.g., EndoScreener and ENDOANGEL) have demonstrated clinical utility as decision-support tools (4, 6, 7). Nevertheless, current approaches have limitations at two levels. Methodologically, most systems rely on image-level fine-grained annotations for training, which are expensive to obtain and subject to considerable interobserver variability. In addition, framewise independent classification does not integrate information across multiple images from the same patient, making it difficult to derive an overall patient-level assessment; moreover, a direct classification output provides limited safety-aware mechanisms to mitigate the clinical consequences of misclassification. From a validation perspective, most studies have been assessed only in single-center datasets, and generalizability across centers remains insufficiently established. Collectively, these limitations hinder the translation of existing systems from the laboratory to real-world clinical practice (8–10).

Despite these advances, evidence remains limited for patient-level AI systems that can learn from pathology-level labels, aggregate information across multiple images from the same examination, and support sensitivity-oriented triage followed by ordered risk ranking. Furthermore, the performance of such systems across institutions with different patient populations and pathological compositions remains insufficiently characterized. To address this knowledge gap, we developed EndoStrat, a patient-level, weakly supervised AI system for gastric lesion screening and risk stratification.

This study aimed to develop and validate the diagnostic and risk-ranking performance of EndoStrat for screening gastric precancerous and early neoplastic lesions. Specifically, we evaluated the sensitivity-oriented triage performance and low-risk assignment rate of the Level-1 module across three validation cohorts; assessed the risk-ranking performance of the Level-2 module among all patients with pathology other than Normal/Superficial, irrespective of Level-1 triage; examined the observed changes in performance after incorporating age and sex; and compared EndoStrat with two comparator methods under identical data splits.

## Methods

### Study design and participants

This retrospective multicenter study included patients who underwent standard white-light upper gastrointestinal endoscopy at three medical centers between January 2017 and December 2024. The inclusion criteria were as follows: (1) age ≥ 18 years; (2) at least 20 archived images per procedure; and (3) a definitive histopathological diagnosis based on biopsy specimens. The exclusion criteria were (1) poor image quality (severe blurring or incomplete mucosal visualization), (2) a history of gastric surgery, and (3) incomplete pathology records.

**Figure 1:**
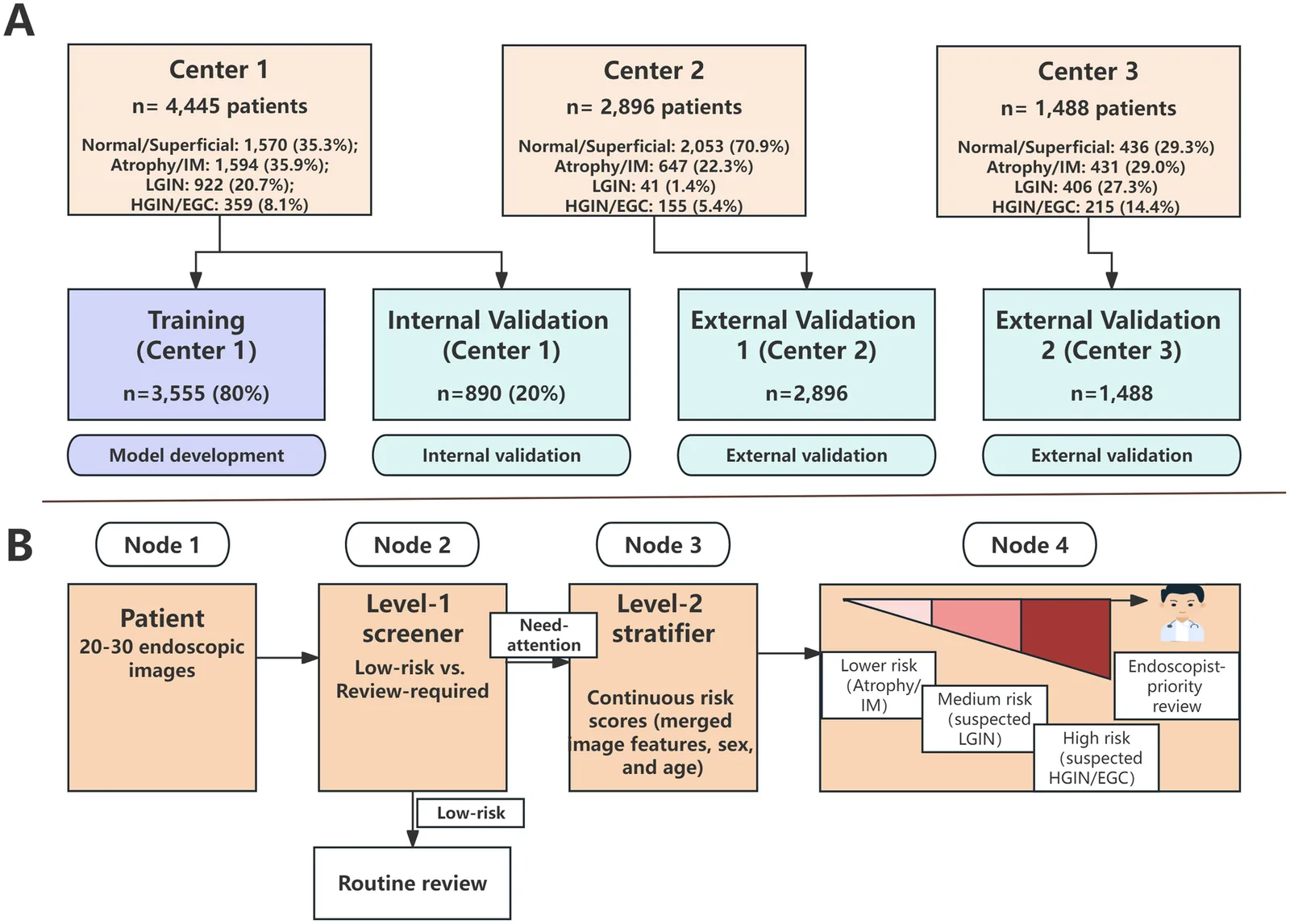
Study overview. **(A)** Patient inclusion and dataset partitioning across three centers. Each center box reports the total number of patients and the distribution across the four pathological categories. Center 1 enrolled 4,445 patients and was stratified by pathology category and randomly split (8:2) into a training cohort (*n* = 3,555) and an internal validation cohort (*n* = 890). All patients from Center 2 (*n* = 2,896) and Center 3 (*n* = 1,488) were used as two independent external validation cohorts (external validation cohort 1 and external validation cohort 2, respectively); these data were not used in any stage of model development. **(B)** Workflow of EndoStrat, a two-stage sequential risk stratification system. Node 1: For each patient, 20–30 white-light endoscopic images are acquired. Node 2: The Level-1 screening module performs binary triage into low-risk vs. review-required patients, and low-risk patients are routed for routine review. Node 3: For review-required patients, the Level-2 stratification module fuses image features with clinical variables (age and sex) and outputs a continuous risk score between 0 and 1. Node 4: The risk score supports priority-based review, with high-risk patients (those with suspected HGIN/EGC) reviewed first.

In total, 8,829 patients were included: 4,445 from Center 1 (The Second Hospital of Hebei Medical University), 2,896 from Center 2 (Cangzhou Central Hospital), and 1,488 from Center 3 (Neiqiu County People's Hospital) (Figure 1A). Patients from Center 1 were split into a training cohort (*n* = 3,555) and an internal validation cohort (*n* = 890) using 8:2 stratified random sampling. Stratification was performed by pathological category to ensure similar class distributions across the two subsets. All patients from Center 2 and Center 3 were used as independent external validation cohort 1 and external validation cohort 2, respectively; these data were not used during any stage of model development (Figure 1A).

The study was approved by the Research Ethics Committee of the Second Hospital of Hebei Medical University (approval no. 2025-R654). The requirement for informed consent was waived because of the retrospective study design.

### Endoscopic image acquisition and preprocessing

Standard white-light upper gastrointestinal endoscopy was performed at all three centers using the same video processing system (CV-290; Olympus Corporation, Tokyo, Japan), with a high-definition video output format of 1080p/1080i. Because the specific gastroscope models were not consistently documented in the retrospective patient-level records, they could not be reliably retrieved for the entire study period. All images included in the analysis were acquired using conventional white-light imaging. For each patient, 20–30 digital images were acquired to cover standard upper gastrointestinal anatomical landmarks; the median number of images per patient was 25 (IQR, 22–28).

Image preprocessing comprises two steps. First, black borders outside the endoscopic field of view were automatically detected and cropped, retaining only valid mucosal regions. Second, Reinhard color normalization was applied to map images from different centers to a common color space, thereby reducing device-related color variation. During training, data augmentation (random flipping, rotation, color jitter, and random grayscale conversion) was used to improve the robustness to cross-center differences in image appearance. All the images were resized to 224 × 224 pixels.

### Pathological diagnosis

All biopsy specimens were interpreted by experienced gastrointestinal pathologists at each center according to the literature (11, 12). Patients were assigned to one of four diagnostic categories: (1) normal mucosa or superficial gastritis (Normal/Superficial); (2) atrophic gastritis or intestinal metaplasia (Atrophy/IM); (3) low-grade intraepithelial neoplasia (LGIN); and (4) high-grade intraepithelial neoplasia, early gastric cancer, or carcinoma *in situ* (collectively, HGIN/EGC). For each patient, the label corresponded to the highest-grade histopathological diagnosis identified during the examination; individual endoscopic images were not annotated.

### Endostrat system architecture

EndoStrat adopts a two-stage, sequential risk stratification architecture (Figure 1B). The Level-1 screening module dichotomizes patients into a low-risk group and a review-required group, enabling the safe triage of low-risk patients. Patients classified as review-required are subsequently processed by the Level-2 risk stratification module, which outputs a continuous risk score ranging from 0 to 1 to support priority-based case review.

In this study, diagnostic labels were available only at the patient level: each patient's set of 20–30 images corresponded to a single diagnostic label, although only a small subset of images might capture the true lesion. We therefore adopted a multiple instance learning (MIL) framework. Specifically, each patient was modeled as a bag (a set of images), and the model learned to identify diagnostically informative images within the bag to generate a patient-level prediction without requiring per-image annotations.

### Image feature extraction and patient-level aggregation

We used a ResNet-50 convolutional neural network pretrained on ImageNet to extract a feature vector from each endoscopic image. Patient-level feature aggregation was performed using the gated attention-based MIL mechanism proposed by Ilse et al (13), rather than a transformer-based MIL architecture. Briefly, the gated attention network calculated an attention score for each image feature, and the resulting attention weights were normalized across all images from the same patient using a softmax function. The patient-level representation was then obtained as the attention-weighted sum of the image features.

### Level-1 screening module

The Level-1 module takes the aggregated patient-level representation as input and outputs a probability between 0 and 1, representing the likelihood that the patient belongs to the review-required group.

To align with the clinical imperative of minimizing missed lesions, we used an asymmetric cost setting during training, penalizing false-negative triage (review-required misclassified as low-risk) five times more than false-positive triage (low-risk misclassified as review-required). This cost-sensitive design promotes high sensitivity, even at the expense of reduced specificity.

The Level-1 operating threshold was determined using the training cohort alone. Because Level 1 was designed as a sensitivity-oriented screening stage, we specified a model-development target of at least 95% sensitivity for the review-required class. Among all candidate thresholds satisfying this sensitivity constraint, we selected the threshold with the highest specificity to correctly triage as many low-risk patients as possible while maintaining the prespecified sensitivity target. This procedure yielded an operating threshold of 0.22. Once selected, the threshold was fixed and applied unchanged to the internal validation cohort and both external validation cohorts, without recalibration or further tuning. The 95% sensitivity target represented a model-development criterion rather than an established clinical safety standard for gastric precancerous lesion screening.

### Level-2 risk stratification module

Among patients classified as review-required by Level 1, the Level-2 module performs refined stratification across three ordered categories (Atrophy/IM → LGIN → HGIN/EGC). Leveraging the ordered progression from mild to severe disease, we formulated this task as ordinal regression and produced a continuous risk score between 0 and 1, with higher scores indicating a higher predicted level of pathological severity. The Level-2 model output was used directly as the continuous risk score; no temperature scaling or additional logit normalization was applied. In contrast to standard multiclass classification, ordinal regression accounts for the ordered relationships among the pathological categories, such that larger discrepancies between the predicted and reference categories receive greater penalties.

Age and sex were incorporated only at Level 2, given their established associations with gastric cancer risk and their utility for refining prioritization among patients with similar endoscopic appearances. We intentionally excluded these clinical variables from Level 1 to reduce the risk that the model would rely on demographic cues for low-risk triage, which could compromise sensitivity in its role as a safety gate.

For Level 2, the backbone network was initialized with the trained Level-1 weights, whereas the attention module was retrained to accommodate the shift in the task objective from “abnormality screening” to “severity stratification.”

### Comparator methods

We included two models as comparator benchmarks under identical data splits.

Image-level classifier: Each endoscopic image was treated as an independent sample and weakly labeled using patient-level diagnosis. At inference, we applied maximum-probability pooling across all images from the same patient, using the maximum predicted probability as the patient-level output. This baseline does not perform patient-level feature aggregation via MIL and was included as a non-MIL comparator benchmark.

Four-class MIL classifier (4-class MIL): This model shares the same backbone and MIL aggregation mechanism as EndoStrat but directly performs four-way classification, without the two-stage workflow design or asymmetric cost setting. It was included as a single-stage patient-level MIL comparator benchmark.

Both comparator models used the same preprocessing, data augmentation, network architecture, and data splits as EndoStrat did, and neither incorporated age or sex to ensure fair comparisons.

### Model training

The backbone network was frozen for the first five epochs to stabilize the newly initialized modules, after which all the parameters were jointly fine-tuned using differential learning rates—a lower rate (1 × 10^−4^) for the pretrained backbone to preserve its learned visual representations and a higher rate (1 × 10^−3^) for the newly initialized attention and classification modules to accelerate convergence. Weighted random sampling was applied to balance the representation of low-risk and review-required patients within each training batch. Models were trained for up to 50 epochs with early stopping based on a composite score (0.7 × sensitivity + 0.3 × AUC) on the internal validation cohort (patience = 10 epochs).

### Statistical analysis

Continuous variables are presented as the mean ± standard deviation or median (interquartile range), and categorical variables as counts and percentages. For Level 1, Normal/Superficial was defined as the negative class, and Atrophy/IM, LGIN, and HGIN/EGC collectively constituted the review-required class. EndoStrat predictions were determined using the exact fixed threshold of 0.22. Level-1 performance was assessed using AUC, sensitivity, specificity, PPV, NPV, likelihood ratios, neoplasia miss rate, and low-risk assignment rate. Neoplasia was defined as LGIN plus HGIN/EGC. Level-2 analyses included all patients with pathology other than Normal/Superficial, irrespective of Level-1 triage, and used the Kruskal–Wallis test, C-index, and QWK. QWK was calculated from the class with the highest predicted probability; risk-score tertile analyses were descriptive. Ninety-five percent confidence intervals for AUC, C-index, and QWK were estimated using 2,000 patient-level bootstrap resamples; Wilson intervals were used for proportions and log-scale intervals for likelihood ratios. Analyses were performed separately within each validation cohort.

## Results

### Baseline characteristics of the study population

In total, 8,829 patients who underwent upper gastrointestinal endoscopy across three centers were included. The study population comprised a training cohort (*n* = 3,555), an internal validation cohort (*n* = 890), external validation cohort 1 (*n* = 2,896), and external validation cohort 2 (*n* = 1,488). The age distributions were similar across cohorts (mean, 47.3–52.5 years). The proportion of male patients ranged from 51.6% to 56.1%, and the median number of endoscopic images per patient was 25 (IQR, 22–28) in all cohorts (Table 1).

**Table 1:** Baseline characteristics of the study population.

Characteristic	Training cohort	Internal validation	External validation 1	External validation 2
Patients, *n*	3,555	890	2,896	1,488
Age, mean ± SD	51.4 ± 13.3	51.4 ± 12.5	47.3 ± 12.7	52.5 ± 13.4
Male, *n* (%)	1,993 (56.1%)	484 (54.4%)	1,494 (51.6%)	821 (55.2%)
Images per patient, median (IQR)	25 (22–28)	25 (22–28)	25 (22–28)	25 (22–28)
Pathological category, *n* (%)				
Normal/Superficial	1,256 (35.3%)	314 (35.3%)	2,053 (70.9%)	436 (29.3%)
Atrophy/IM	1,275 (35.9%)	319 (35.8%)	647 (22.3%)	431 (29.0%)
LGIN	737 (20.7%)	185 (20.8%)	41 (1.4%)	406 (27.3%)
HGIN/EGC	287 (8.1%)	72 (8.1%)	155 (5.4%)	215 (14.4%)

The pathological composition differed across cohorts. Pathology distributions were similar between the training and internal validation cohorts, with neoplasia (LGIN + HGIN/EGC) accounting for approximately 28.8% of patients in both cohorts. In external validation cohort 1, Normal/Superficial cases accounted for 70.9% of patients, and the prevalence of neoplasia was 6.8%. In external validation cohort 2, the prevalence of neoplasia was 41.7%, including 14.4% of patients with HGIN/EGC (Table 1).

### Diagnostic performance of the level-1 screening module

EndoStrat follows a two-stage sequential design. Level 1 dichotomizes all patients, triaging low-risk cases to standard review while routing review-required cases to Level 2 for further stratification. To assess the added value of the MIL framework and the two-stage design, we evaluated two comparators: (i) an image-level baseline that performs per-image classification and applies maximum-probability pooling to obtain a patient-level output and (ii) a 4-class MIL model that shares the same architecture and aggregation mechanism as EndoStrat but directly outputs four-class predictions without the two-stage workflow.

**Figure 2:**
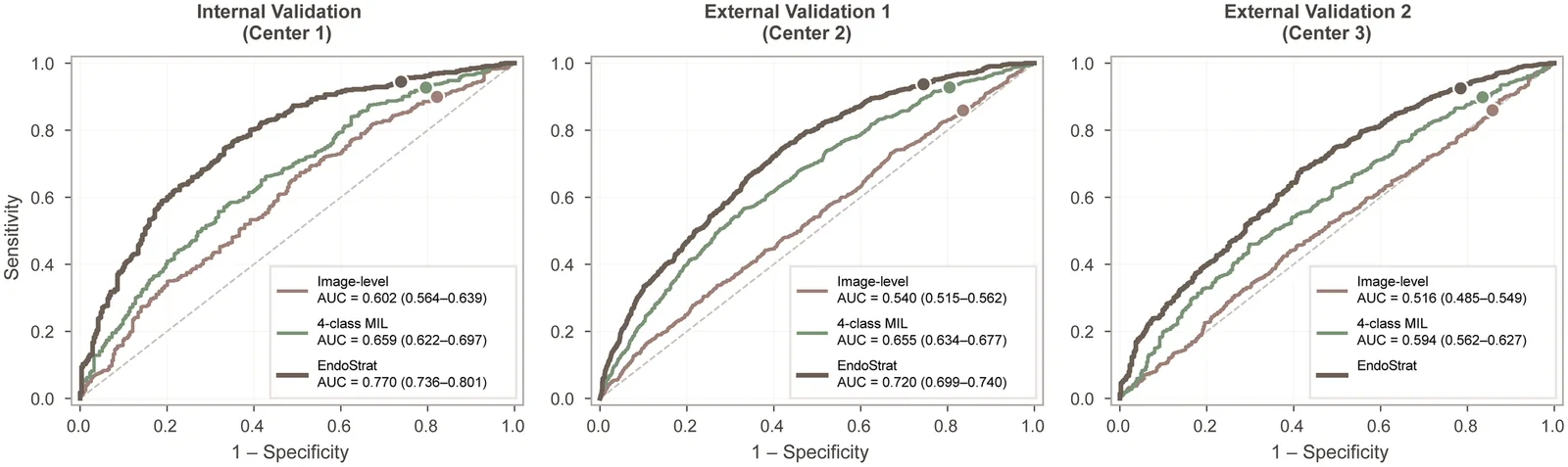
ROC curves of the level-1 safety screening gates across the three validation cohorts. Receiver operating characteristic (ROC) curves of the three methods in the internal validation cohort, external validation cohort 1, and external validation cohort 2. Within each panel, ROC curves are shown for the image-level baseline (dashed), 4-class MIL (dash-dotted), and EndoStrat (solid). The area under the curve (AUC) and 95% confidence interval (bootstrap, 2,000 resamples) are reported in the legend. Dots denote operating points (sensitivity and 1−specificity) under a fixed decision threshold determined on the training cohort to achieve an overall sensitivity ≥95% at the Level-1 gate, which was then applied unchanged to all validation cohorts.

Across the three validation cohorts, EndoStrat Level 1 achieved AUCs of 0.770 (95% CI, 0.736–0.801), 0.720 (95% CI, 0.699–0.740), and 0.674 (95% CI, 0.644–0.705), respectively. The corresponding AUCs were 0.602 (95% CI, 0.564–0.639), 0.540 (95% CI, 0.515–0.562), and 0.516 (95% CI, 0.485–0.549) for the image-level baseline and 0.659 (95% CI, 0.622–0.697), 0.655 (95% CI, 0.634–0.677), and 0.594 (95% CI, 0.562–0.627) for the 4-class MIL comparator (Figure 2 and Table 2). At the exact fixed operating threshold of 0.22, EndoStrat achieved sensitivities of 0.944 (95% CI, 0.923–0.960), 0.938 (95% CI, 0.920–0.953), and 0.930 (95% CI, 0.913–0.944) in the internal validation cohort, external validation cohort 1, and external validation cohort 2, respectively. The corresponding specificities were 0.261 (95% CI, 0.216–0.312), 0.251 (95% CI, 0.233–0.270), and 0.211 (95% CI, 0.175–0.252) (Table 2).

**Table 2:** Performance of the EndoStrat level-1 screening module across the validation cohorts.

Metric	Internal validation	External validation 1	External validation 2
AUC (95% CI)	0.770 (0.736–0.801)	0.720 (0.699–0.740)	0.674 (0.644–0.705)
Sensitivity (95% CI)	0.944 (0.923–0.960)	0.938 (0.920–0.953)	0.930 (0.913–0.944)
Specificity (95% CI)	0.261 (0.216–0.312)	0.251 (0.233–0.270)	0.211 (0.175–0.252)
PPV (95% CI)	0.701 (0.668–0.732)	0.340 (0.321–0.359)	0.740 (0.715–0.763)
NPV (95% CI)	0.719 (0.631–0.794)	0.908 (0.882–0.929)	0.554 (0.478–0.628)
LR+ (95% CI)	1.278 (1.193–1.369)	1.253 (1.215–1.291)	1.178 (1.119–1.240)
LR− (95% CI)	0.213 (0.145–0.313)	0.246 (0.187–0.323)	0.333 (0.251–0.443)
Neoplasia miss rate (95% CI)	5.8% (3.6%–9.4%); 15/257	6.1% (3.5%–10.4%); 12/196	8.1% (6.2%–10.5%); 50/621
Overall low-risk assignment rate (95% CI)	12.8% (10.8%–15.2%); 114/890	19.6% (18.2%–21.1%); 567/2,896	11.2% (9.7%–12.9%); 166/1,488

### Neoplasia miss rates and low-risk assignment at level 1

**Figure 3:**
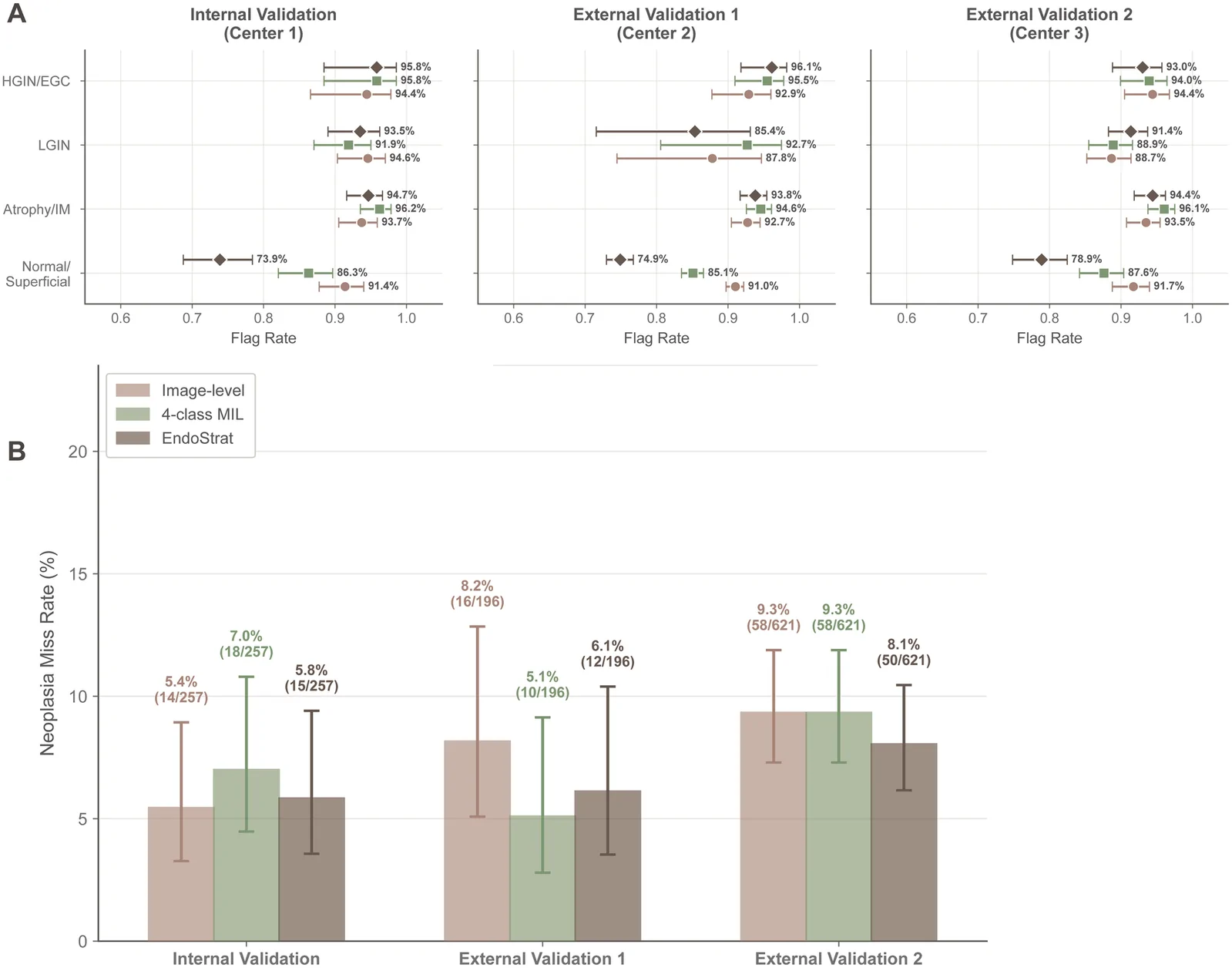
Subtype-specific flag rates and neoplasia miss rates of the level-1 safety screening gate. **(A)** Flag expression rates (i.e., the proportion classified as review-required by Level 1) stratified by pathological subtype. The *x*-axis shows the flag rate, and the *y*-axis lists (top to bottom) HGIN/EGC, LGIN, Atrophy/IM, and Normal/Superficial. The methods are indicated by the following markers: circles (image-level baseline), squares (4-class MIL), and diamonds (EndoStrat). The error bars indicate Wilson 95% confidence intervals, and the point estimates are annotated. For Normal/Superficial samples, the flag rate corresponds to the false-positive rate (1−specificity); lower values indicate better specificity. **(B)** Neoplasia miss rates computed by combining LGIN and HGIN/EGC. The three validation cohorts are shown side by side; within each cohort, the bars represent the three methods. Bar labels report the miss rate and the absolute number of missed cases/total neoplasia cases; error bars indicate Wilson 95% confidence intervals.

At the exact fixed operating threshold of 0.22, the neoplasia miss rates of EndoStrat were 5.8% (15/257; 95% CI, 3.6%–9.4%), 6.1% (12/196; 95% CI, 3.5%–10.4%), and 8.1% (50/621; 95% CI, 6.2%–10.5%) in the internal validation cohort, external validation cohort 1, and external validation cohort 2, respectively. Among patients with Normal/Superficial pathology, 26.1% (95% CI, 21.6%–31.2%), 25.1% (95% CI, 23.3%–27.0%), and 21.1% (95% CI, 17.5%–25.2%) were classified as low risk in the three cohorts, respectively. When calculated across all patients, the corresponding overall low-risk assignment rates were 12.8% (114/890; 95% CI, 10.8%–15.2%), 19.6% (567/2,896; 95% CI, 18.2%–21.1%), and 11.2% (166/1,488; 95% CI, 9.7%–12.9%), respectively (Figure 3 and Table 2).

### Risk stratification performance of the level-2 module

**Figure 4:**
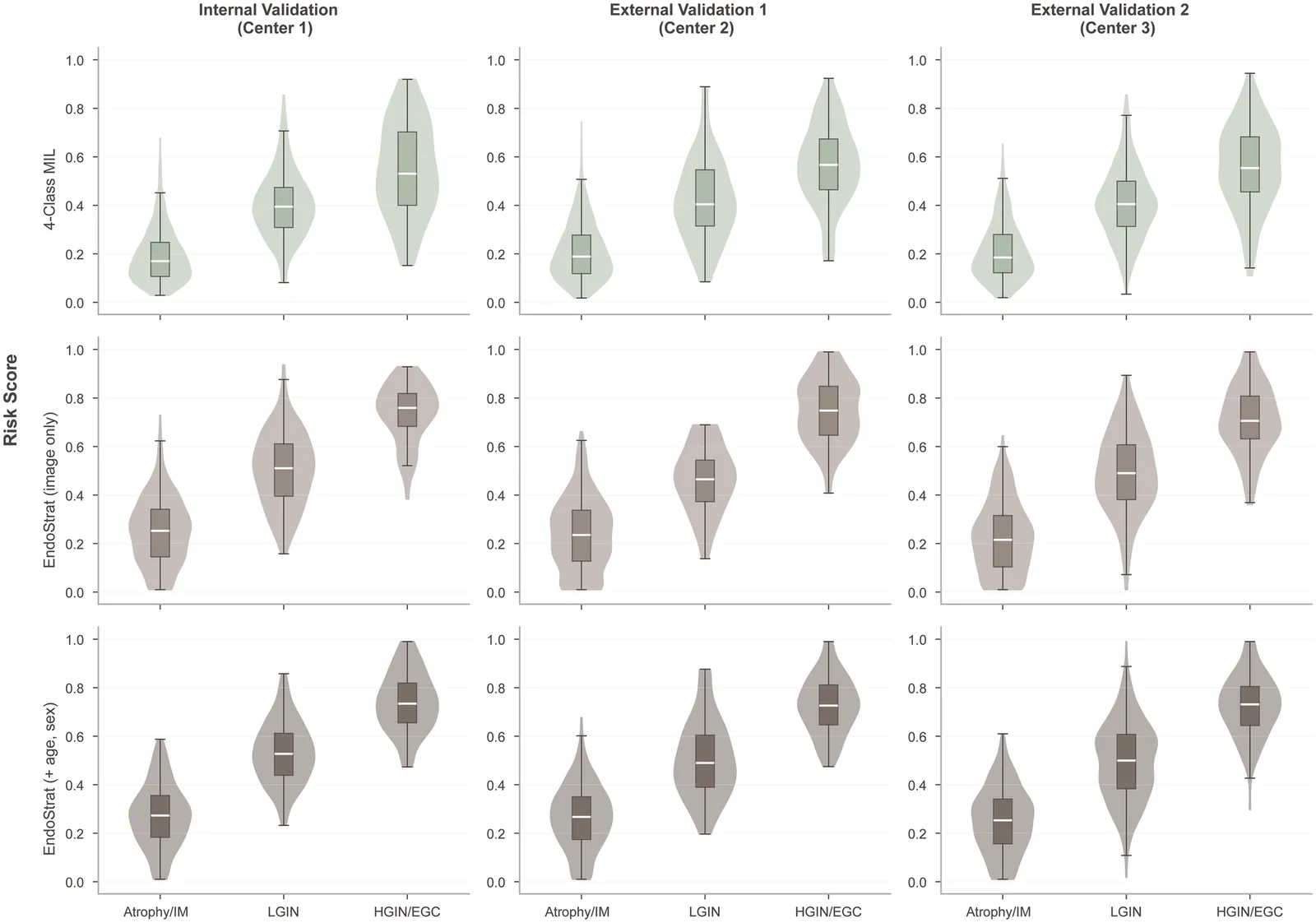
Level-2 risk score distributions in patients excluding normal/superficial. Risk score distributions across three model variants (rows) and three validation cohorts (columns). Rows, top to bottom: 4-class MIL [risk index = P(LGIN) × 0.5 + P(HGIN/EGC) × 1.0], EndoStrat (image only), and EndoStrat with age and sex. Violin plots show score density distributions with embedded boxplots indicating the median and interquartile range. The analyses included all non-Normal/Superficial patients (internal validation, *n* = 576; external validation cohort 1, *n* = 843; external validation cohort 2, *n* = 1,052).

To evaluate the risk-ranking performance of the Level-2 module independently of Level-1 triage, the analysis included all patients excluding those in the Normal/Superficial category (internal validation cohort, *n* = 576; external validation cohort 1, *n* = 843; external validation cohort 2, *n* = 1,052). Across the three cohorts, the median risk scores ranged from 0.25 to 0.27 for Atrophy/IM, from 0.49 to 0.53 for LGIN, and were approximately 0.73 for HGIN/EGC. Differences among the three pathological categories were statistically significant in all cohorts (all Kruskal–Wallis *p* < 0.001; Figure 4 and Table 3).

**Table 3:** Performance of the endoStrat level-2 risk-stratification module across the validation cohorts.

Metric	Internal validation (image only)	Internal validation (+age, sex)	External validation 1 (image only)	External validation 1 (+age, sex)	External validation 2 (image only)	External validation 2 (+age, sex)
Patients other than Normal/Superficial, n	576	576	843	843	1,052	1,052
C-index (95% CI)	0.916 (0.896–0.933)	0.937 (0.923–0.951)	0.966 (0.952–0.978)	0.967 (0.955–0.979)	0.903 (0.890–0.916)	0.907 (0.894–0.918)
QWK (95% CI)	0.694 (0.647–0.737)	0.675 (0.627–0.717)	0.834 (0.791–0.872)	0.861 (0.822–0.896)	0.662 (0.627–0.696)	0.704 (0.673–0.733)
Risk score, median (IQR)						
Atrophy/IM	0.252 (0.145–0.341)	0.273 (0.183–0.356)	0.235 (0.127–0.337)	0.267 (0.174–0.351)	0.215 (0.104–0.315)	0.253 (0.156–0.341)
LGIN	0.511 (0.395–0.611)	0.527 (0.439–0.612)	0.465 (0.373–0.544)	0.490 (0.390–0.605)	0.490 (0.381–0.607)	0.499 (0.383–0.608)
HGIN/EGC	0.760 (0.683–0.818)	0.734 (0.655–0.819)	0.748 (0.646–0.848)	0.726 (0.647–0.811)	0.706 (0.632–0.808)	0.731 (0.644–0.805)
Kruskal–Wallis *p* value	<0.001	<0.001	<0.001	<0.001	<0.001	<0.001

After age and sex were incorporated, the C-index changed from 0.916 (95% CI, 0.896–0.933) to 0.937 (95% CI, 0.923–0.951) in the internal validation cohort, from 0.966 (95% CI, 0.952–0.978) to 0.967 (95% CI, 0.955–0.979) in external validation cohort 1, and from 0.903 (95% CI, 0.890–0.916) to 0.907 (95% CI, 0.894–0.918) in external validation cohort 2 (Figure 4 and Table 3).

**Figure 5:**
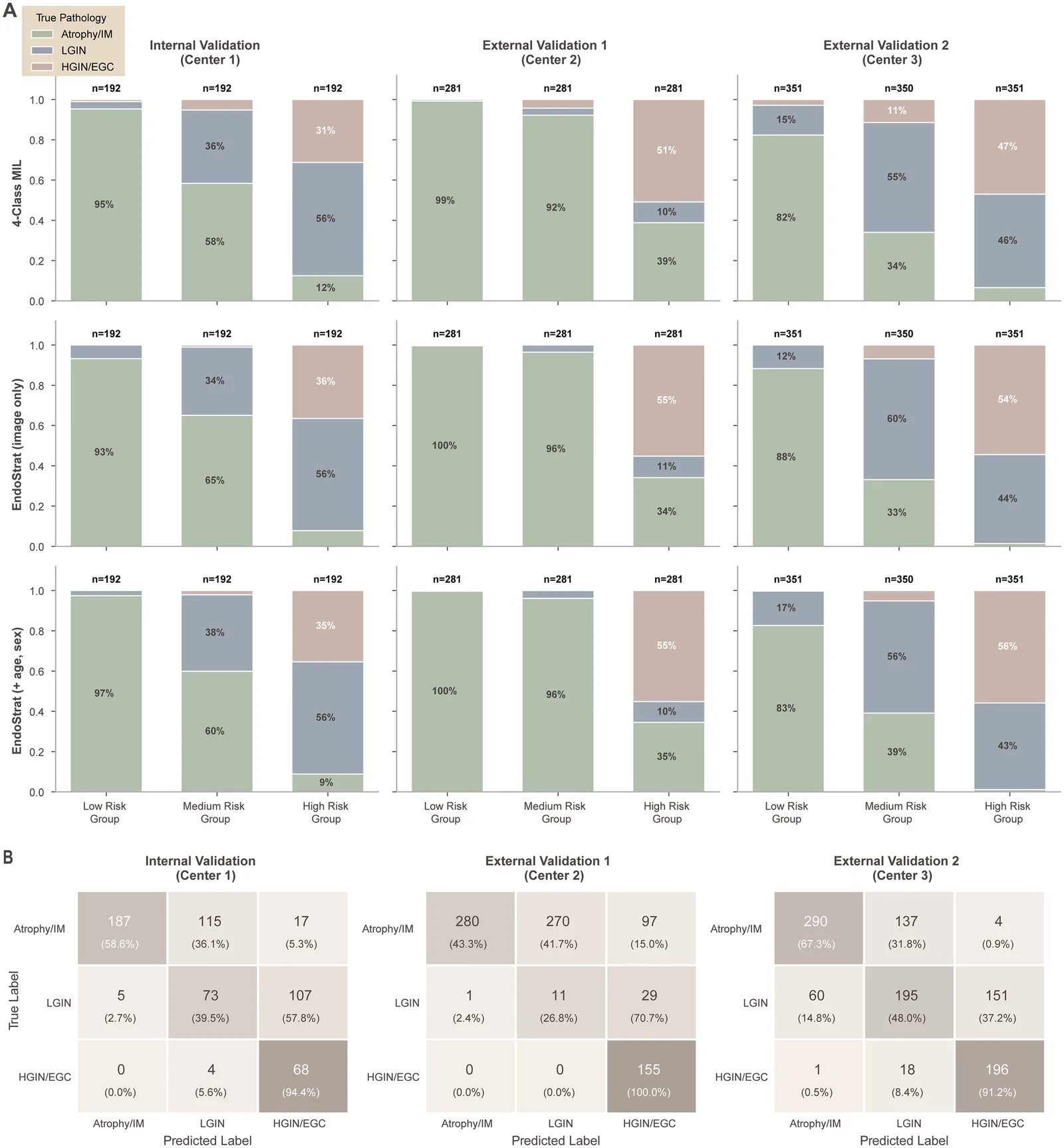
Clinical consistency analyses for level-2 risk stratification. **(A)** Pathological composition by risk score tertiles. Rows correspond to the three model variants (top to bottom: 4-class MIL, EndoStrat image-only, and EndoStrat with clinical variables); columns correspond to the three validation cohorts. Stacked bars show the proportions of Atrophy/IM (green), LGIN (blue-gray), and HGIN/EGC (rose) within each tertile, with sample sizes annotated above the bars. **(B)** Confusion matrices of the final EndoStrat model incorporating age and sex across the three validation cohorts. The predicted categories were assigned descriptively according to risk-score tertiles (lowest: Atrophy/IM; middle: LGIN; highest: HGIN/EGC). Cells report absolute counts and row percentages. Darkened diagonal cells indicate correct classifications. All analyses included patients with pathology other than Normal/Superficial. The tertile-based classifications shown in the confusion matrices were descriptive and were not used to calculate the QWK values reported in Table 3.

In the descriptive tertile-based analysis, the proportion of Atrophy/IM was highest in the lowest risk-score tertile, whereas the proportion of HGIN/EGC was highest in the highest tertile (Figure 5A). For the final EndoStrat model incorporating age and sex, the proportions of HGIN/EGC cases assigned to the highest risk-score tertile were 94.4% (95% CI, 86.6%–97.8%), 100.0% (95% CI, 97.6%–100.0%), and 91.2% (95% CI, 86.6%–94.3%) in the internal validation cohort, external validation cohort 1, and external validation cohort 2, respectively. Separately, based on the three-class predictions defined by the highest predicted class probability, QWK changed from 0.694 (95% CI, 0.647–0.737) to 0.675 (95% CI, 0.627–0.717) in the internal validation cohort, from 0.834 (95% CI, 0.791–0.872) to 0.861 (95% CI, 0.822–0.896) in external validation cohort 1, and from 0.662 (95% CI, 0.627–0.696) to 0.704 (95% CI, 0.673–0.733) in external validation cohort 2 after age and sex were incorporated (Table 3).

### Representative cases

**Figure 6:**
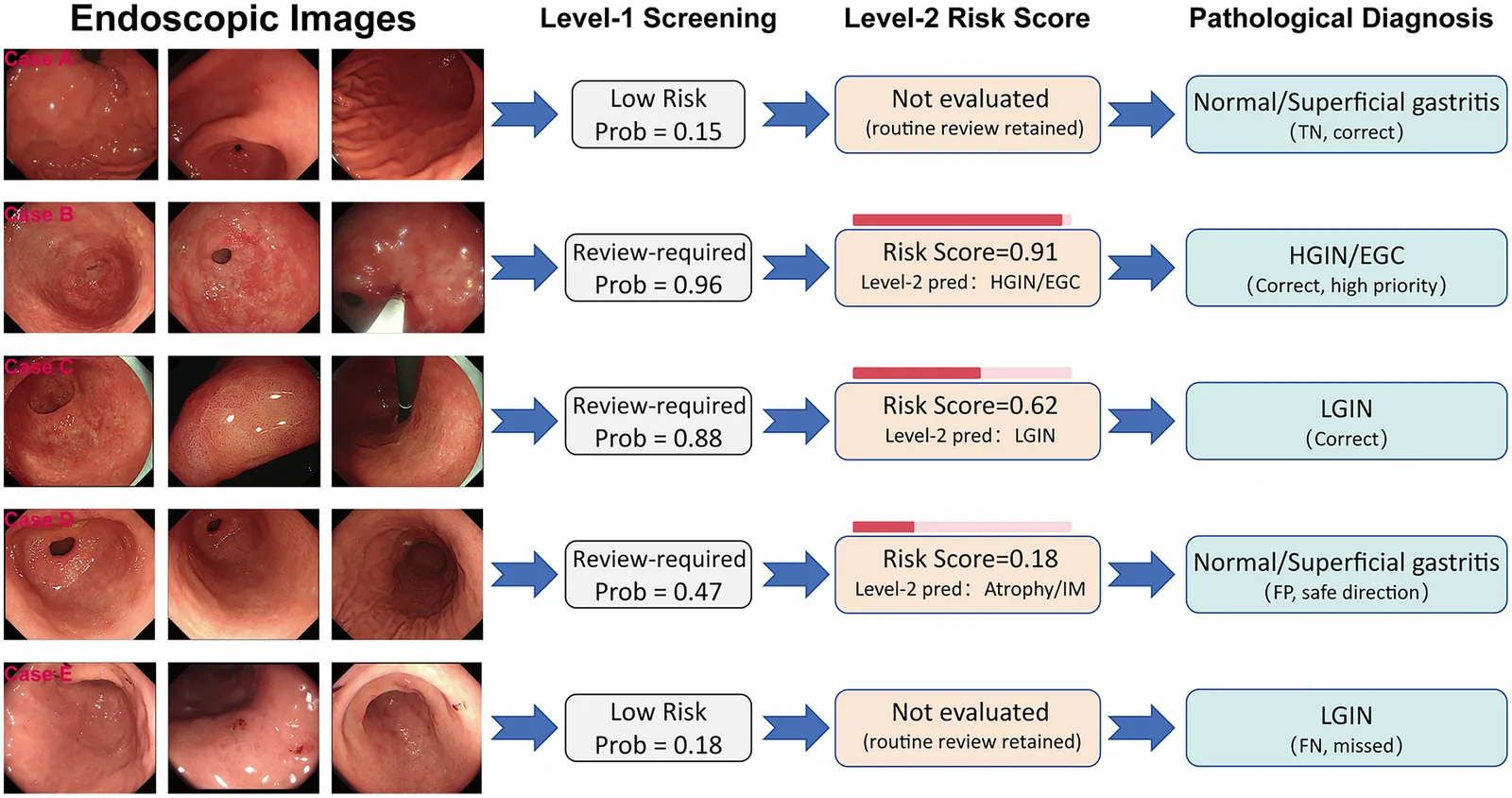
Representative cases illustrating EndoStrat's two-stage workflow. Five representative cases are shown to illustrate the clinical decision process of the two-stage EndoStrat system. Each row represents one patient and, from left to right, includes endoscopic images (2–3 representative images), the Level-1 output (including the predicted probability), the Level-2 risk score (including the predicted class; shown only for patients classified as review-required at Level 1), and the pathology ground truth together with the system output. The filled length and color intensity of the Level-2 risk score bar are proportional to the risk score.

Figure 6 presents five representative cases illustrating the outputs of the two-level EndoStrat system. Case A, diagnosed as Normal/Superficial gastritis, was classified as low risk at Level 1 (probability, 0.15) and did not undergo Level-2 stratification. Case B, diagnosed as HGIN/EGC, was classified as review-required at Level 1 (probability, 0.96) and assigned a Level-2 risk score of 0.91, with HGIN/EGC as the predicted category. Case C, diagnosed as LGIN, was classified as review-required at Level 1 (probability, 0.88) and assigned a Level-2 risk score of 0.62, with LGIN as the predicted category. Case D, diagnosed as Normal/Superficial gastritis, was classified as review-required at Level 1 (probability, 0.47) and assigned a Level-2 risk score of 0.18, with Atrophy/IM as the predicted category. Case E, diagnosed as LGIN, was classified as low risk at Level 1 (probability, 0.18), was not processed by the Level-2 module, and remained assigned to routine clinician review.

## Discussion

In this retrospective multicenter study, EndoStrat showed sensitivity-oriented Level-1 triage performance and an ordered Level-2 risk-score gradient across Atrophy/IM, LGIN, and HGIN/EGC. The findings support further investigation of patient-level, weakly supervised risk stratification for post-procedural review prioritization, while its clinical effects and applicability beyond the evaluated settings remain to be established.

At Level 1, the HGIN/EGC flag rate was 93.0% or higher across all three validation cohorts, and the patient-level neoplasia miss rate ranged from 5.8% to 8.1% at the exact fixed operating threshold of 0.22. Among patients with Normal/Superficial pathology, 21.1%–26.1% were classified as low risk, whereas the overall low-risk assignment rates across the full validation cohorts ranged from 11.2% to 19.6%. These values describe the proportions assigned to the low-risk pathway under the evaluated retrospective conditions; the actual effect on endoscopists' workload was not directly measured. Direct numerical comparison with adenoma miss rates reported in colonoscopy studies is difficult because the lesion definitions, imaging workflows, and denominators differ substantially (14, 15). Moreover, no consensus patient-level miss-rate threshold has been established for gastric precancerous lesion screening (16, 17).

Several factors may have contributed to the missed cases. Under patient-level weak supervision, each set of 20–30 images was assigned a single pathological label, although only a small proportion of the images may have depicted the lesion. Small or flat LGIN and HGIN/EGC lesions, or lesions showing only subtle changes in mucosal color or texture, may therefore have received insufficient weight during patient-level aggregation. Retrospectively archived images may also have provided incomplete mucosal coverage or failed to capture the most diagnostically informative views. Importantly, low-risk assignment at Level 1 changes only the priority of case review. These patients are not excluded from clinical assessment and continue to undergo routine review by endoscopists. Clinician review therefore remains an essential safety safeguard following model-based triage, and EndoStrat is intended to support review prioritization rather than independently exclude lesions or replace endoscopists.

Existing AI-assisted endoscopy systems, such as EndoScreener (18, 19) and ENDOANGEL (20–22), primarily provide real-time lesion detection or quality control during endoscopy. In contrast, EndoStrat aggregates multiple endoscopic images at the patient level and provides two-stage risk stratification to support post-procedural case-review prioritization. EndoStrat may therefore complement these existing systems; however, because no head-to-head comparison was performed, their relative performance cannot be determined from the present study.

At Level 2, the continuous risk score increased monotonically across the ordered subtypes Atrophy/IM, LGIN, and HGIN/EGC, supporting the use of ordinal regression to model the intrinsically ordered clinical spectrum of disease severity. After age and sex were incorporated, the C-index increased modestly in all three cohorts, while QWK increased in the two external cohorts but decreased slightly in the internal validation cohort. Because these analyses included all patients with pathology other than Normal/Superficial irrespective of Level-1 classification, the reported C-index and QWK represent module-level rather than end-to-end performance. In the two-stage workflow, only patients classified as review-required at Level 1 proceed to Level 2; therefore, overall workflow performance also depends on Level-1 triage. These findings describe the performance of the combined model but do not establish the independent contribution of either clinical variable (2). Finally, the greater difficulty in distinguishing LGIN from HGIN/EGC may partially reflect the known subjectivity and interobserver variability in grading these lesions on histopathology, in addition to the limitations inherent to image-based prediction (15, 23).

EndoStrat showed higher observed discrimination and risk-ranking performance than the comparator models in the evaluated cohorts. However, because the models differed in multiple methodological components, these comparisons do not isolate the respective contributions of MIL aggregation, the sequential two-level design, or cost-sensitive training. Unlike the image-level baseline, the MIL framework aggregates information from multiple images acquired from the same patient without requiring image-level annotations. EndoStrat also separates Level-1 screening from Level-2 risk stratification and uses a cost-sensitive objective at Level 1. These components were designed to support sensitivity-oriented triage and subsequent risk ranking, but their individual effects were not evaluated separately. At the prespecified threshold, the majority of neoplastic cases were assigned to the review-required group; however, the extent to which this result was attributable specifically to the two-level architecture could not be determined from the present analyses.

Several limitations should be acknowledged. First, this was a retrospective study, and the effects of EndoStrat on clinical decision-making, workflow efficiency, and patient outcomes have not been prospectively evaluated in routine clinical practice. Second, we did not conduct a controlled comparison between EndoStrat-assisted and unassisted endoscopist assessment; therefore, its incremental benefit for endoscopists with different levels of experience remains unknown. Future prospective, multicenter, real-world studies should compare EndoStrat-assisted assessment with independent endoscopist assessment and evaluate its effects on diagnostic accuracy, case-review time, clinical workflow, and patient outcomes. Third, the reliance on patient-level weak supervision limits spatial interpretability and precludes precise lesion localization. Because expert-annotated image-level or lesion-level reference standards were unavailable, we could not determine whether images receiving high attention weights corresponded to the actual lesion locations. Moreover, attention weights indicate the relative contribution of individual images to the model output but should not be interpreted as validated lesion localization or causal explanations. Future studies should systematically assess the agreement between high-attention images and expert-annotated lesion locations and include endoscopist-involved evaluations of model interpretability before such visualizations are used for clinical decision support. Fourth, all participating centers were located in Hebei Province, China, served populations with broadly similar geographic and ethnic backgrounds, and used the same Olympus CV-290 video processing system. However, the specific gastroscope models could not be reliably retrieved from the retrospective patient-level records, limiting the assessment of equipment-related heterogeneity. Consequently, generalizability across geographic regions, ethnic populations, healthcare systems, and endoscopic devices remains to be established before widespread clinical implementation.

In summary, EndoStrat showed sensitivity-oriented Level-1 triage performance and ordered Level-2 risk scores across three retrospective cohorts. These findings support further prospective evaluation of patient-level AI-assisted review prioritization. Its effects on clinician performance, workflow efficiency, and patient outcomes, together with its generalizability across populations, healthcare systems, and endoscopic platforms, must be established before clinical implementation.

## Data Availability

The raw data supporting the conclusions of this article will be made available by the authors, without undue reservation.
